# *Verbascum gongliuensis* (Scrophulariaceae), a new species of Yili, Xinjiang, China

**DOI:** 10.3897/phytokeys.280.201229

**Published:** 2026-10-14

**Authors:** Yuhao Luo, Wenbin Xu

**Affiliations:** 1 The College of Life Sciences, Shihezi University, Shihezi, Xinjiang, China The College of Life Sciences, Shihezi University Shihezi China

**Keywords:** New species, *
Verbascum
*, Yili

## Abstract

*Verbascum gongliuensis* Y.H. Luo & W.B. Xu (Scrophulariaceae), is described as a new species from Yili, Xinjiang, China. Based on careful comparison with the Flora of China, Flora Xinjiangensis, Flora of Kazakhstan, Flora of Turkey and the East Aegean Islands, and the Flora of the USSR, it is confirmed as a new species of Verbascum. Its threat category was assessed according to IUCN (2001) criteria. A comprehensive description of the new species is provided, accompanied by detailed photographs, a distribution map, and its IUCN conservation status.

## Introduction

The genus *Verbascum* L. is placed in Scrophulariaceae and is mainly distributed across Eurasia, comprising more than 360 species. Six species are recorded from China, one of which is incompletely known or of doubtful status. The genus has its highest species richness in West Asia and Eastern Europe. Turkey contains 254 species of *Verbascum*, of which 193 are endemic (Huber-Morath 1978), which makes Turkey the country with the greatest *Verbascum* diversity worldwide.

The taxonomy of *Verbascum* has undergone multiple revisions (Alzahrani et al. 2025), and new species continue to be discovered (Fırat 2015; Zografidis 2016). Linnaeus (1753), in Species Plantarum, separated the genus into *Celsia* (with four stamens) and *Verbascum* (with five stamens) based on stamen number. Bentham (1846) divided *Celsia* and *Verbascum* into two sections, *Thapsus* Benth. and *Lychnitis* Benth., based on anther morphology: the former with anthers decurrent along the filament, the latter with reniform anthers. Murbeck (1925) further subdivided *Celsia* according to seed morphology into *Bothrospermae* Murb. and *Aulacospermae* Murb.; the former was further divided into *Nefflea* Benth. (all anthers reniform) and *Arcturus* Benth. (the two anterior anthers reduced). Later, Murbeck (1933) divided *Verbascum* based on seed morphology into *Bothrospermae* Murb. and *Aulacospermae* Murb., with Bothrospermae further split into *Fasciculata* Murb. (flowers clustered) and *Singuliflora* Murb. (flowers solitary). Ferguson (1971) and Huber-Morath (1973) subsequently merged Celsia into *Verbascum*. Current molecular phylogenetic studies (Remal 2014; Ghahremaninejad et al. 2015; Sotoodeh 2015; Dong et al. 2022; Alzahrani et al. 2024) strongly support the inclusion of *Celsia* within *Verbascum*.

Using stamen number as the primary diagnostic character, the Flora of the USSR recognized two separate genera, Celsia and *Verbascum*, with four species of Celsia and 46 species of *Verbascum*. Nine *Verbascum* species have been documented in Kazakhstan. In China, *Verbascum* is mainly distributed in western regions, with the greatest diversity in the Xinjiang Uygur Autonomous Region. A total of six species are known from China, five of which occur in Xinjiang: *V. thapsus* L., *V. blattaria* L., *V. songaricum* Schrenk, *V. phoeniceum* L., and *V. chaixii* Vill. subsp. orientale Hayek (Flora Reipublicae Popularis Sinicae, Zhong 1979; Flora Xinjiangensis, Pan and Nurbay 2004).

During field surveys conducted in Gongliu County, Yili, Xinjiang, between 2023 and 2026, a *Verbascum* species entirely covered with glandular hairs was discovered. At first glance, it superficially resembles *V. macrocarpum* and *V. blattaria*, but important differences were observed (e.g., in corolla, calyx, capsule, plant height, glandular hairs, bracts, and basal leaves). After collecting specimens, it was found to possess a combination of characters not present in any other *Verbascum* species; therefore, it is described here as a new species, *Verbascum gongliuensis*.

## Materials and methods

The specimens were cross-checked using the key provided by Huber-Morath (1978) and various relevant publications, including the Flora of China (Zhong 1979), Flora Xinjiangensis (Pan and Nurbay 2004), Flora of Kazakhstan (Semiotrocheva 1965), and the Flora of the USSR (Fedchenko 1955). They were also examined and compared with specimens deposited in the Herbarium of Shihezi University (SHI) and with records available in online databases including iPlant (2025), Plants of the World Online (POWO 2025), Plant Photo Bank of China (2025), and the Global Biodiversity Information Facility (GBIF 2026). The specimens were further compared with recently published accounts of new *Verbascum* species (Karavelioğulları et al. 2011, 2023; Fırat 2015; Sotoodeh et al. 2015, 2022; Zografidis 2016; Firat 2022; Zografidis et al. 2022; Duran 2023). Plants and their diagnostic details were photographed with a digital camera, geographic coordinates were recorded with a GPS unit, and floral and fruit structures were dissected and examined under a magnifying lens and a stereomicroscope. The threat category was assessed according to the International Union for Conservation of Nature (IUCN) Red List Categories and Criteria, version 3.1 (IUCN 2001).

## Taxonomic treatment

### Verbascum gongliuensis

Taxon classificationPlantaeLamialesScrophulariaceae

Y.H.Luo & W.B.Xu sp. nov.

3BDF471D-4990-5164-A0D5-0E5884A146C8

urn:lsid:ipni.org:names:77380708-1

Figs 1, 2, 3

#### Type.

China • Xinjiang, Yili Kazakh Autonomous Prefecture, Gongliu County, Kuerdening; 43°08'46"N, 82°51'41"E; alt. 1614.48 m; alpine forest edge; 30 Jul. 2024; *Wenbin Xu & Yuhao Luo 4546* (holotype: SHI!; isotype: SHI!).

**Figure 1.**
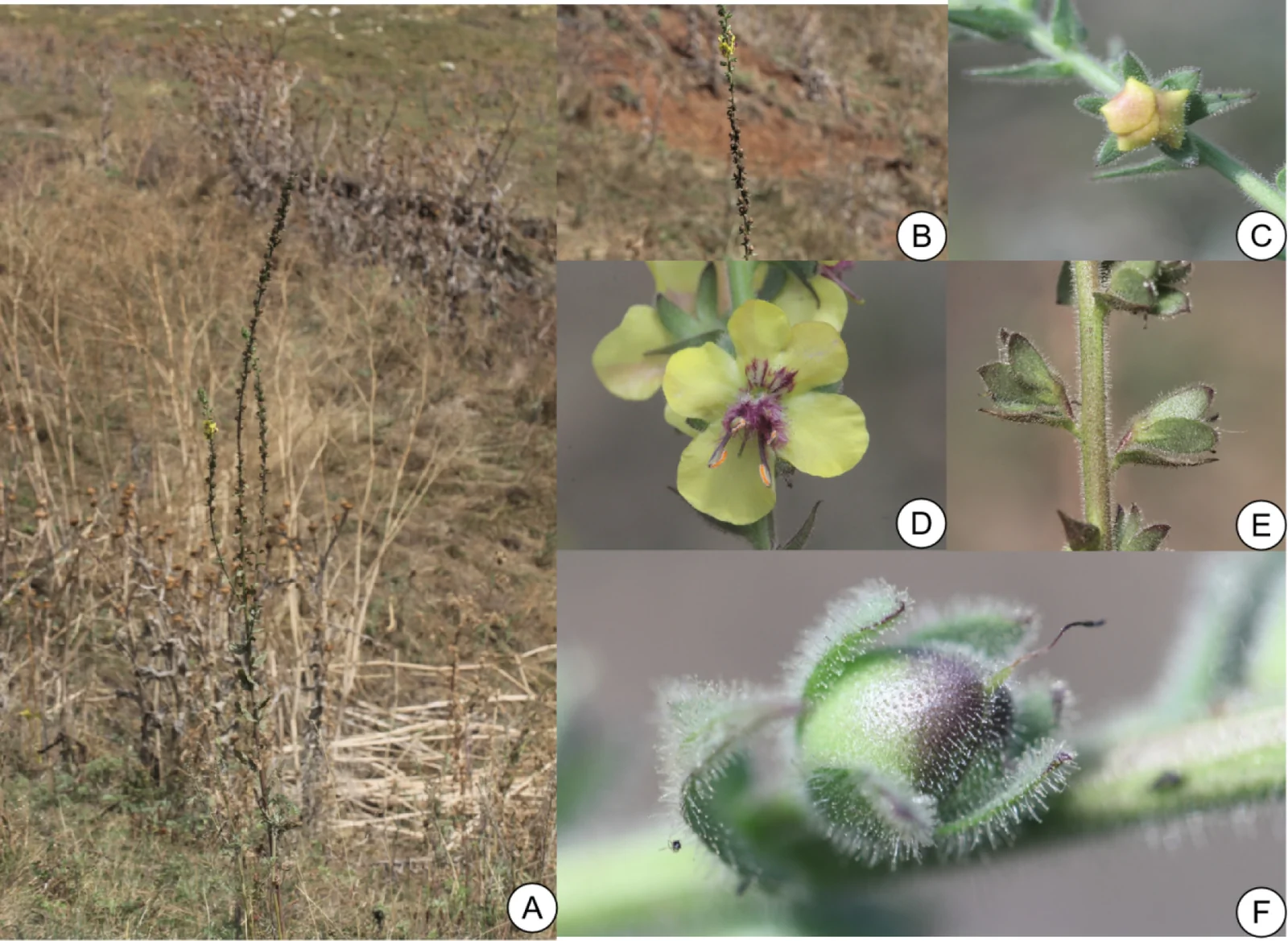
*Verbascum gongliuensis*. **A**. Plant and habitat; **B**. Inflorescence; **C**. Flower bud; **D**. Corolla; **E**. Calyx and bracts; **F**. Capsule.

**Figure 2.**
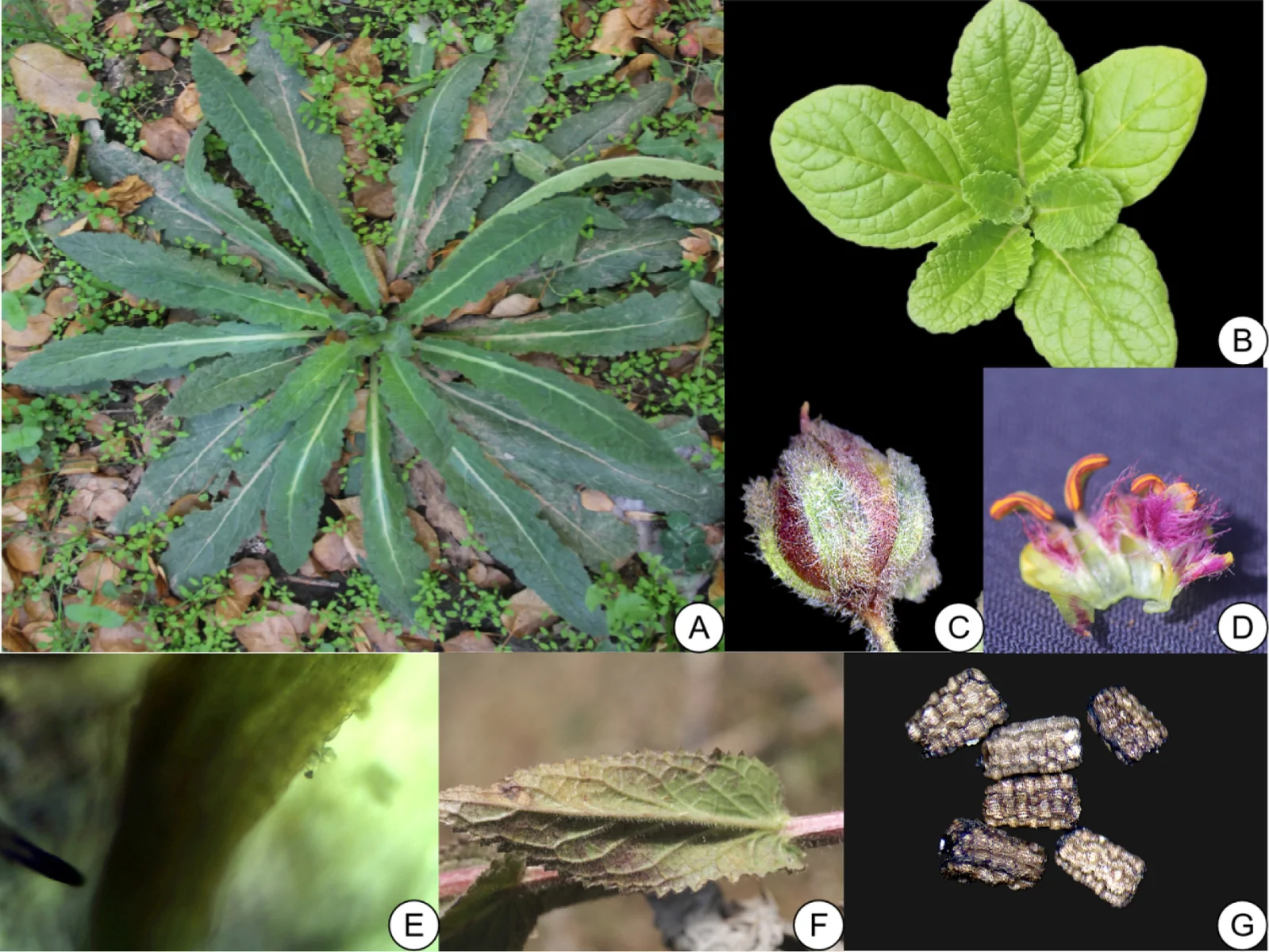
*Verbascum gongliuensis*. **A**. Basal leaves; **B**. Basal leaves (young); **C**. Capsule; **D**. Dissected flower (stamina 4); **E**. Filaments; **F**. Cauline leaves; **G**. Seeds.

**Figure 3.**
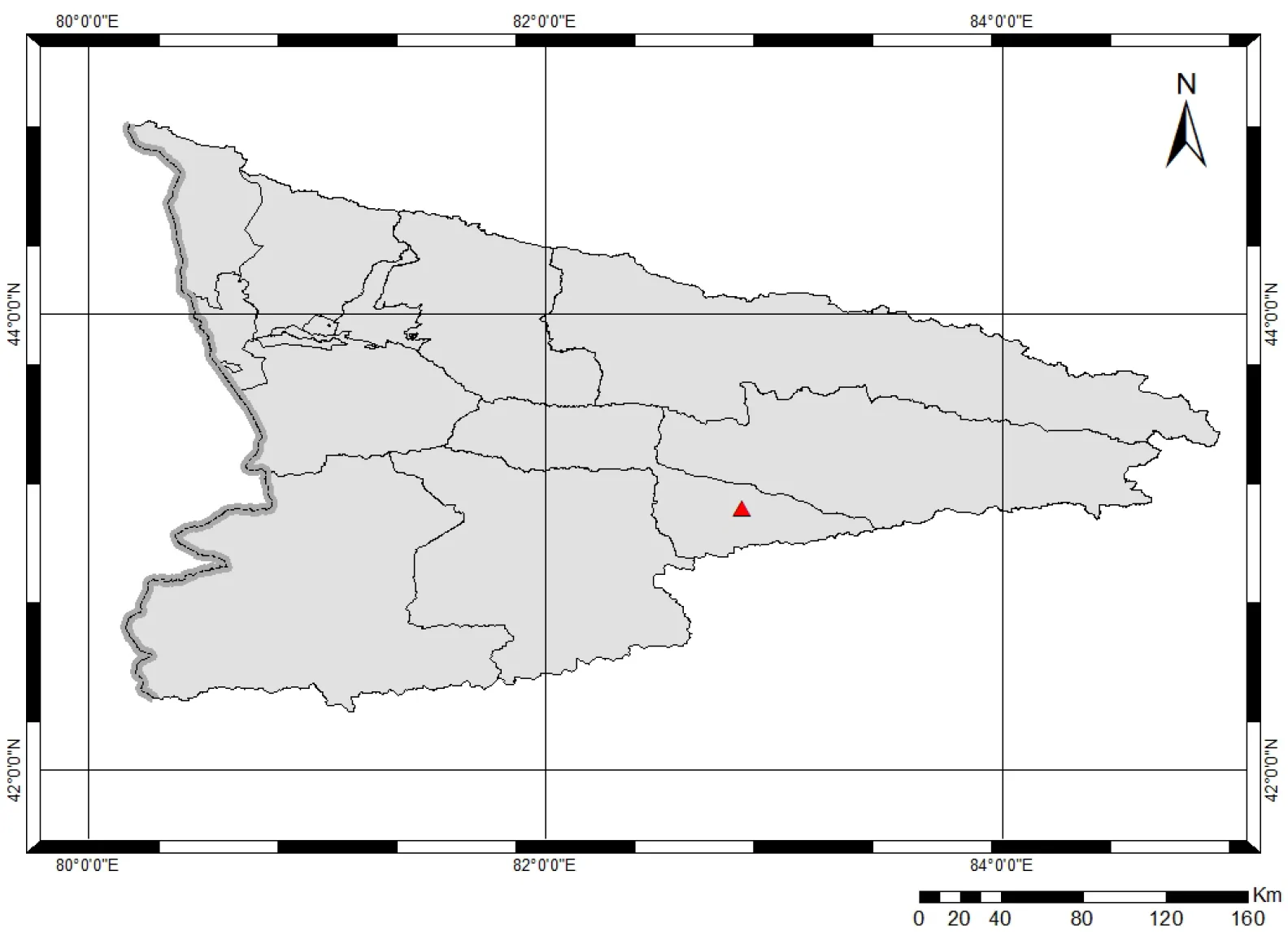
Distribution map of *Verbascum gongliuensis*.

#### Diagnosis.

*Verbascum gongliuensis* is morphologically most similar to *V. macrocarpum*, from which it differs by its bracts being shorter than the flowers (vs. longer than the flowers), a smaller corolla, a denser glandular indumentum, and a capsule that conspicuously exceeds the persistent calyx. It is further distinguished from *V. ponticum* by the smaller corolla (18–23 mm vs. 26–33 mm) and by the exclusively glandular indumentum. From *V. bornmuellerianum*, it can be separated by the two anterior filaments being glabrous near the apex (vs. filament hairs violet and extending right up to the anthers). From *V. zerdust*, it is readily distinguished by having 5 stamens (rarely 4) rather than 4.

#### Description.

Biennial herb. Stem unbranched or branched from the middle, 30–150 cm tall, the whole plant densely covered with long-stalked glandular hairs. Leaves simple, alternate; basal leaves ovate, elliptic to lanceolate, margins bicrenate, the teeth undulate, both surfaces densely glandular-hairy, more densely so along veins abaxially, 5–35 cm long, 3–6 cm wide, petiole 3–6 cm long; cauline leaves sessile, oblong-ovate to triangular-ovate, margins dentate, both surfaces densely glandular-hairy, 3–8 cm long, 1–3 cm wide. Inflorescence a raceme, simple or few-branched; flowers solitary; bracts lanceolate to triangular-lanceolate, densely glandular-hairy, 0.5–1.5 cm long; pedicels slightly thickened in fruit, 2–5 mm long. Calyx laterally compressed after flowering, densely glandular-hairy, almost divided to the base, lobes lanceolate to elliptic-lanceolate, ca. 0.5 cm long. Corolla yellow, densely glandular-hairy outside; upper two lobes with purple and white appendages at base, 18–23 mm in diameter; corolla lobes unequal, the upper two slightly smaller, the lower three larger, 5–12 mm long. Stamens 4 or 5, 4–6 mm long; filaments with purple woolly hairs, unequal in length; filaments of the two lower longer stamens glabrous near apex, with sessile glandular hairs at base; anthers laterally compressed, curved, narrowly reniform, decurrent at base, two larger and three or two smaller, the larger 2–3 mm long, the smaller 0.5–1.5 mm long. Capsule exceeding calyx, ovoid-globose, densely glandular-hairy, 6–10 mm long, 6–12 mm wide, apex with a short beak ca. 1.5 mm long. Flowering June–August, fruiting July–September. Seeds cylindrical, 0.5–1 mm long, with longitudinal undulate striations.

#### Specimens examined (type material).

**Figure 4.**
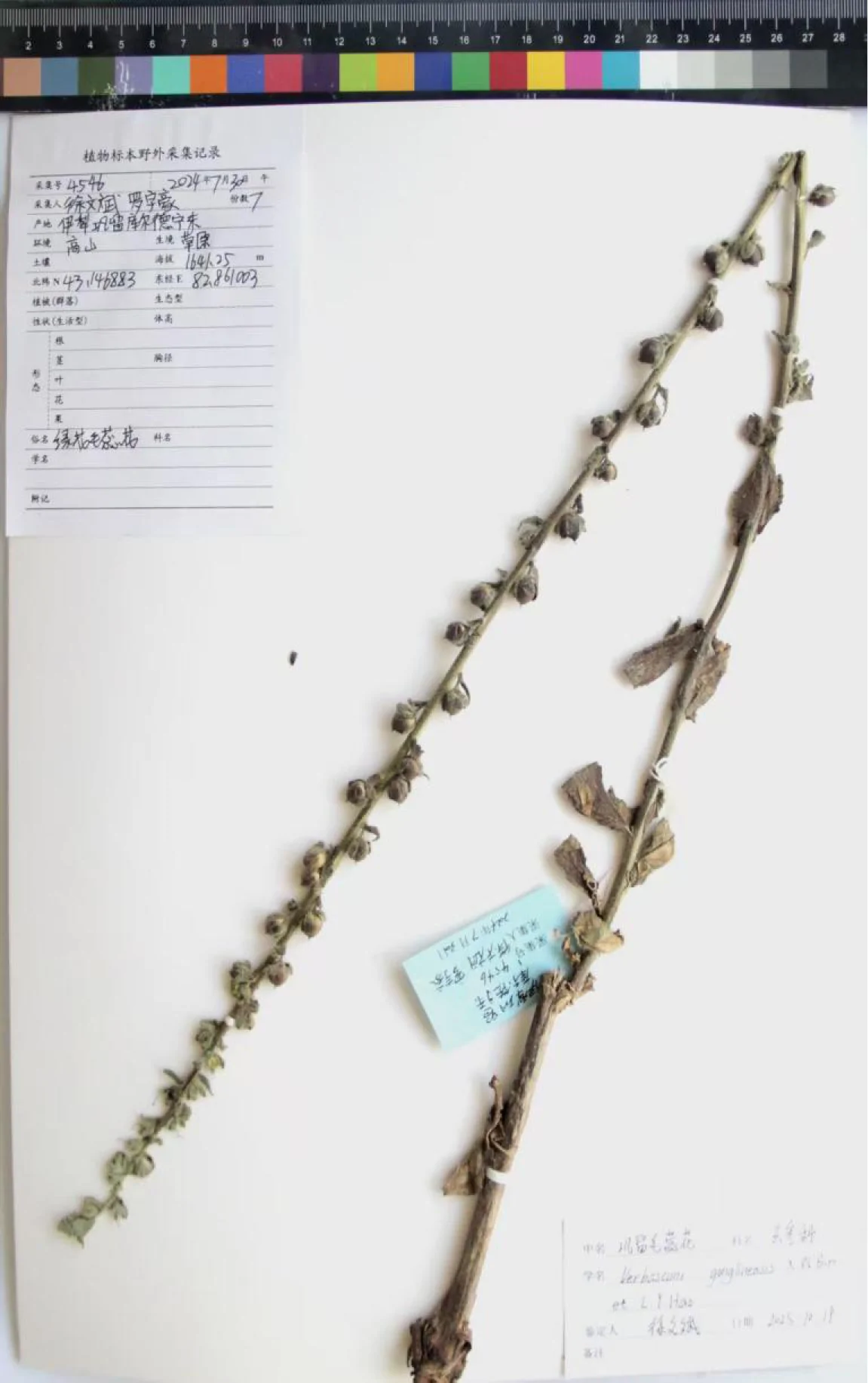
Holotype specimen of *Verbascum gongliuensis* sp. nov.

China • Xinjiang: Gongliu County, Kuerdening; alpine grassland; alt. 1614.48 m; 30 Jul. 2024; Wenbin Xu & Yuhao Luo 4546 (SHI) (Fig. 4).

#### Red list assessment.

*Verbascum gongliuensis* is currently known from a single location, with an estimated extent of occurrence of less than 20 km^2^ and an estimated population size of fewer than 1000 mature individuals. The habitat is significantly threatened by grazing and tourism activities, which are projected to cause a continuing decline in the number of mature individuals. Therefore, the species is assessed as Vulnerable VU [D1+2] according to IUCN (2001) criteria.

## Discussion

*Verbascum gongliuensis* differs morphologically from all Verbascum species recorded in the Flora of the USSR and the Flora of Turkey. Its closest relatives may be *V. macrocarpum* and *V. blattaria*, but it differs from *V. macrocarpum* and *V. zerdust* in many morphological characters (e.g., corolla, calyx, bracts, stamens, basal leaves, cauline leaves, pedicels, capsules, etc.) (Table 1). Geographically, *V. gongliuensis* occurs in alpine steppe at 1600 m, whereas *V. blattaria* inhabits lower-elevation riverbanks and marshes. Furthermore, based on our cultivation of different Verbascum species, *V. gongliuensis* may prefer humid and low-temperature environments, while *V. blattaria* is more tolerant of high temperatures and strong sunlight. Although the habitat of *V. macrocarpum* is similar to that of *V. gongliuensis*, the nearest record of *V. macrocarpum* is from the southern basin of Lake Alakol in Kazakhstan, approximately 800 km from Kuerdening. In addition, Kuerdening is located in the central Tianshan Mountains, forming a geographical isolation from *V. macrocarpum*, which has led to differences in corolla size, bract length, stamen size, and other characters.

**Table 1.** Diagnostic morphological comparison among *Verbascum gongliuensis*, *V. macrocarpum* and *V. zerdust*.

**Character**	***V. gongliuensis* sp. nov**.	***V. macrocarpum***	***V. zerdust***
Stem	Unbranched or branched from middle, 30–150 cm tall, densely covered with glandular hairs of unequal length	Robust, unbranched; 50–200 cm tall; densely covered with glandular hairs of unequal length	Sometimes branched in upper part; 40–80 cm tall; upper part densely viscose-villous from long articulate hairs, sparsely so below, rarely short articulate-hairy, mostly glandular throughout
Basal leaves	5–35 × 3–8 cm, ovate to oblanceolate, apex obtuse, crenate; petiole 3–6 cm long	6–20 × 2.5–8 cm, lanceolate-oblong to ovate, apex obtuse, coarsely crenate; petiole 2–6 cm long	8–15 × 3–7 cm, broadly ovate, cordate, irregularly dentate, acute; petiole 8–11 cm long
Cauline leaves	Oblong-ovate to triangular-ovate, dentate, densely glandular-hairy	Smaller; upper leaves ovate to lanceolate, acuminate, base subcordate, sparsely glandular-hairy on both surfaces	Ovate to ovate-lanceolate, cordate to semiamplexicaul, crenate-dentate, acute; upper leaves sessile
Inflorescence	Raceme, many-flowered	Raceme, many-flowered	Cylindrical, dense, many-flowered
Bracts	Longer than pedicels, shorter than flowers, lanceolate to triangular-lanceolate; upper bracts entire, lower ones dentate below	Longer than flowers, ovate-triangular to lanceolate-ovate; lower ones dentate	Lower bracts cordate-ovate, cordate, crenulate, acute; upper bracts ovate-lanceolate, entire, acuminate, distinctly 3–5-veined, densely articulate-hairy on both surfaces
Pedicels	2–5 mm long	3–8 mm long	1–3 mm long
Calyx	3–8 mm long, divided almost to base; lobes flat, lanceolate to elliptic-lanceolate, apex shortly acute	7–10 mm long; lobes unequal, narrowly oblong to elliptic-oblong, apex obtuse or mucronate	7–9 mm long, glandular and articulate-hairy; lobes ovate-lanceolate, acute
Corolla	Yellow, 18–23 mm; upper two lobes with purplish and white papillae	Yellow, 25–30 mm, without pellucid glands, outside with articulate glandular hairs	Actinomorphic, rotate, 20–30 mm diam., outside glandular and articulate-hairy
Stamens	4 or 5; filaments with purple woolly hairs; anthers decurrent at base; two anterior filaments glabrous near apex; anthers 0.5–3 mm long, decurrent	5, rarely 4; filament hairs purple-violet; two anterior filaments glabrous near apex; anthers 3–4 mm long, decurrent	4; filament hairs purple-violet, extending right up to anthers; two anterior anthers decurrent, 2–5 mm long
Capsule	6–10 × 6–12 mm, ovoid-globose, glandular-hairy, exceeding calyx	7–9 mm, subglobose, glandular-hairy, scarcely exceeding calyx	6–8 × 5–7 mm, ovate, rarely articulate-hairy

### Key to the species of *Verbascum* in China

**Table:** 

1	Flowers solitary in the axil of each bract; capsule distinctly longer than the persistent calyx; plants glandular-hairy	**2**
–	Flowers 2 to several fascicled in the axil of each bract; capsule equaling or only slightly longer than the persistent calyx; plants stellate-hairy	**5**
2	Corolla purple; capsule surface with raised reticulate ridges	***V. phoeniceum***
–	Corolla yellow; capsule surface without raised reticulate ridges	**3**
3	Stamens 4; leaves lyrate-parted	***V. chinense***
–	Stamens 5; leaves undivided, crenate	**4**
4	Capsule 5–7 mm; glandular hair stalks shorter	***V. blattaria***
–	Capsule 6–10 mm; glandular hair stalks longer	***V. gongliuensis***
5	Inflorescence a dense cylindrical spike	***V. thapsus***
–	Inflorescence an open panicle	**6**
6	Plants sparsely hairy, cauline leaves green, adaxially subglabrous or very sparsely hairy	**V. chaixii subsp. orientale**
–	Plants densely covered with thick greyish-white tomentum, cauline leaves not green adaxially	***V. songaricum***

## Supplementary Material

XML Treatment for Verbascum gongliuensis

## Additional information

### Artificial Intelligence (AI) use

The authors accept full responsibility for the content of the manuscript, including the disclosure of any use of AI.

Regarding the use of AI in the preparation of this manuscript, the authors declare the following: DeepSeek, Doubao.

**Used for**: Language, style and writing, Figures, tables and charts. The DeepSeek-V3 model was used for English translation of the manuscript, and the DouBao model was used to remove non-subjective parts from the figures. AI models did not participate in the creation of the main content, scientific design, data analysis, taxonomic description, or conclusion formulation of this paper. All final content was reviewed, verified, and approved by the authors.

### Author ORCIDs

Y. Luo https://orcid.org/0009-0006-0279-0685

### Data availability

All of the data that support the findings of this study are available in the main text.
